# Historical Reflection of Food Processing and the Role of Legumes as Part of a Healthy Balanced Diet

**DOI:** 10.3390/foods9081056

**Published:** 2020-08-04

**Authors:** Patricia Huebbe, Gerald Rimbach

**Affiliations:** Institute of Human Nutrition and Food Science, University of Kiel, 24118 Kiel, Germany; rimbach@foodsci.uni-kiel.de

**Keywords:** diet-related health risk, unhealthy food tax, dietary guidelines

## Abstract

The purpose of food processing has changed over time. High-intensity industrially processed food often exhibits higher concentrations of added sugar, salt, higher energy, and lower micronutrient density than does similar food or meals prepared at home from raw or minimally processed food. Viewing the evolution of food processing from history, one could make out three major transitions related to human socioeconomic changes. The first transition was marked by the change from hunting and gathering to settled societies with agriculture and livestock farming. The second and third transitions were associated with the Industrial Revolution and with market liberalization, global trade and automation, respectively. The next major transition that will influence food processing and shape human nutrition may include the exploitation of sustainable and efficient protein and food sources that will ensure high-quality food production for the growing world population. Apart from novel food sources, traditional food such as legumes and pulses likewise exhibit great potential to contribute to a healthy balanced diet. The promotion of legumes should be intensified in public dietary guidelines because their consumption is rather low in high-income countries and increasingly displaced as a traditional staple by industrially processed food in low- to middle-income countries.

## 1. Invention of Food Processing

**Figure 1:**
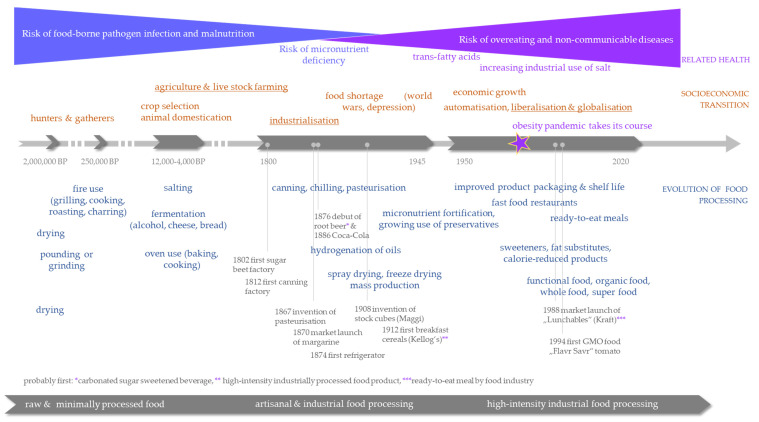
Time-dependent evolution of food processing associated with major transitions in human socioeconomic conditions.

Food processing is not an invention of modern times but presumably has already been employed by the early archaic humans of the Early and Middle Pleistocene [1,2,3]. At that time, the first species of the Homo genus emerged more or less consecutively (*Homo habilis* and *Homo erectus*) and were characterized by increasing brain and body size compared to the preceding Australopithecines. The anatomical evolution was accompanied by an increased resting metabolic energy demand and required increased dietary energy yield [4]. It is supposed that apart from a higher proportion of animal-based food, such as meat, the application of non-thermal food processing, such as pounding, cutting, grinding or sun-drying, contributed to the increased dietary quality of archaic humans [1,5]. Regardless of the food that would have been on their menu, it was certainly ‘minimally processed’ rather than raw [6]. Raw food requires anatomical specification (e.g., of the jaw or digestive tract), which seems absent in humans; in contrast, adaption to prepared food is suggested [7]. From a certain point on, thermal processing became critical for human energy demands. Clearly, with the use of fire, raw meat and carrion became edible, safe and more palatable. Additionally, the dietary quality of grains, tubers, legumes and other plant foods benefits from cooking, including the gelatinization of starch or the denaturation of antinutrient ingredients [1]. There are indications of the opportunistic use of fire dating back to 1.8 million years ago, although the interpretation of the African and Eastern Asian archeologic excavations is not undisputed [8,9,10,11,12]. However, it seems widely accepted that the regular use of fire (with the ability and will of fire production) at northern latitudes emerged somewhat 400,000–300,000 years ago [7,13,14]. From that approximate time, the Neanderthals inhabited wide parts of southwestern and Central Europe and Asia and are considered to have been sophisticated fire users. They made use of a variety of plant foods, such as grains and legumes, through cooking, complementing their otherwise likely animal-based diet [15]. Genetic evidence, however, suggests that cooking preceded the divergence of the modern from the archaic humans, which would have occurred much earlier than archaeological evidence proposes [2]. Importantly, among the plant remains excavated from Middle Paleolithic sites in the Near East (dating 50,000–60,000 before present (BP)), legumes were found most, indicating that they were highly consumed plant foods at that time [16]. Over time, the processing of food has changed, from the use of Paleolithic stone tools, sun-drying and bonfire grilling to industrial roller mills, spray drying and electrical pressure-cooking (Figure 1).

## 2. The Purpose and Technology of Food Processing are Changing

Over the course of time, three major socioeconomic transitions are thought to have driven the evolution of food processing most. The first event may have been the transition from hunter-gatherers to pastoralism, followed by the transition into agriculture and livestock farming approximately 15,000 to 10,000 years ago. As a result, the human diet changed towards a higher emphasis on dairy, cereals and grains. The seed that was used likely underwent selection for several thousands of years [17], probably as long as the domestication of goats and sheep took. In addition to grain cultivation and early milk production, the traditions of fermenting food, baking bread and making cheese emerged. The oldest archaeological evidence of brewing beer and making bread are suggested to be 13,000 and 14,000 years old, respectively [18,19], while cheesemaking began in Europe approximately 8000 years ago [20]. In addition to crop cultivation and farming, the storage and preservation of food have gained importance. During the Neolithic period, prehistoric European and Chinese societies established the first saltworks (5000–6000 BP), and from then on, salt became the most important food preservative for several millennia and a valuable commodity.

Bread has been an important staple food throughout the European Middle Ages, and therefore, milling technology made progress during that period. Since the time of the Roman Empire, white bread has been a privilege of the rich and powerful, while rural and less privileged people consumed primarily dark bread out of mixed grains (e.g., wheat, barley) and legumes mixed with vegetables [21].

The second major socioeconomic transition bringing food processing to the next level was the Industrial Revolution during the 18th and 19th centuries [22]. Major milestones included the generation and use of electricity, the mass production of steel and the transition from hand manufacturing to steam machines and internal combustion engines. Improvements in food processing included the introduction of steam and rolling mills for refined flour production and the enhancement of food preservation techniques [23]. In the 1860s, Louis Pasteur proved that food spoilage can be attributed to the presence of microbes and established a novel preservation method using mild heating that has been named after him (pasteurization).

However, the refinement of grain milling, including the separation and elimination of the germ to increase the storage stability of flour, contributed to severe micronutrient deficiencies in the population in the late 19th and early 20th centuries [23] until the existence and essentiality of micronutrients was established. Since the 1940s, flour enrichment that reintroduces lost vitamins and minerals during grain processing (e.g., thiamine, niacin, folic acid or iron) has been applied. This gave rise to further industrial food fortification efforts that also had partly questionable value. For instance, the vitamin fortification of unhealthy snacks or beer, potentially allowing for advertisements claiming health benefits, has been banned by national authorities. In contrast, micronutrient enrichment and fortification may promote public health and has been shown to specifically contribute to the iron, folate, vitamin A and D intake of US residents [24]. However, general food fortification is also under critical debate, as individual subgroups of the population could be at risk of exceeding the upper level of the recommended intake of certain nutrients [24].

## 3. Demand for Convenient Food is Rising

During the 20th century, the purpose of food processing has changed, starting from improving the safety, shelf life or nutritional value to increasing the convenience and palatability of food. Along with the growing economic prosperity after the two world wars, the purchase of industrially processed food requiring minimal preparation at home continued to increase in industrialized high-income countries. This may be attributed in part to the growing urbanization and number of working women. Consequently, there was less time left over for cooking, and traditional knowledge on the preparation of certain foods was lost. As the desire for convenient and pre-processed food rose, the food industry met the growing demand by intensifying the grade of processing and developing so-called ready-to-heat or ready-to-eat food or dishes. In addition, a transformation of the food industry took place, separating the site of production from the processing sector. Transportation routes, as well as the storage capacity of whole products and isolated ingredients, could be extended. From then on, modern food had to cover a growing path from the original producer to industrial processing to retail. The “in-between processing” of food (between producer and retail) gained massive importance [23].

Advances in broiler chicken breeding led to a worldwide boom in broiler production (chickens raised exclusively for meat production) in the 1950s and to increased poultry consumption in the following decades. The wide distribution of refrigerators and deep freezers in private households was likewise associated with the greater purchase and consumption of perishable products, such as milk, eggs, cheese and meat [25]. Consequently, dietary intake of animal-based food and fat has been increasing since the beginning of the 20th century, reaching a peak in the early 1970s. Dietary fat intake has been risen at the expense of carbohydrates, a transition that became noticeable through the declining consumption of potatoes and grains in the UK since the late 19th century [25]. However, conditions changed when the overconsumption of saturated fat and cholesterol, particularly from red meat and sausages, was deemed to be at least partly causative in cardiovascular mortality in the early 1960s [26]. Consequently, the dietary use of butter or lard was restrained in private households and the food industry in favor of vegetable oils [27], especially from soybeans in the USA [28].

In addition to replacement by vegetable oils, the limitation of animal-based fats was associated with a rise in carbohydrate consumption (by 10% of total energy) [29], particularly in the form of refined sugars, such as sugar-sweetened beverages and frosted breakfast cereals [27]. Since the 1960s, the average intake of refined sugars has increased in high-income countries in contrast to the intake of dietary fiber, a phenomenon that has also been observed in middle- and low-income countries with a certain time lag [27]. The growing consumption of sugar-sweetened beverages and industrially highly processed food is associated with the third transition in human nutrition. It was facilitated by global trade liberalization, market opening and transnationally operating giant corporations selling tobacco and alcoholic beverages in addition to processed food. The growing sales of industrially processed food and sugar-sweetened beverages preceded the worldwide sharp increase in average body mass index (BMI), waist circumference and obesity rates, suggesting a dramatic decline in nutritional quality [30,31,32,33,34,35,36,37,38,39,40]. There are many examples of high- to middle- and low-income countries in which the opening of the food market to global trading affected local dietary habits by superseding traditional staples through the introduction of high-intensity industrially processed food [27,37,38,41,42,43,44]. Legumes and pulses give a good example of how food processing impacts human diet. The major importance of the rise in high-intensity industrial food processing for individual food choices and traditional food culture emphasizing legumes and pulses will be presented in the following.

## 4. Historical Change of Legumes and Pulses Consumption

**Figure 2:**
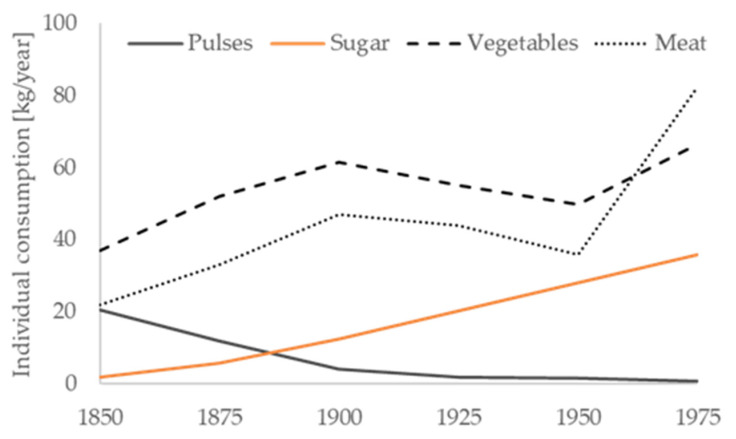
Nutrition transition in Germany, with special emphasis on pulses, refined sugar, vegetables and meat. Data on food consumption are adopted from Teuteberg (1979) [49].

Over the course of time, pulses have been a major staple food for several millennia, and there is a growing body of evidence that grains and pulses contributed to the daily diet of early humans not less than 60,000 to 40,000 years ago [15,16]. Along with other crops, legumes were domesticated during the Neolithic period and have been widely cultivated for at least 4000 years until the present time. Especially in the southern and western regions of Europe, pulses have been very commonly consumed throughout history due to their nourishing and filling characteristics. Legumes exhibit good storage properties after drying and many agronomic advantages including rhizobial nitrogen fixation [45]. Isotope analyses reconstructing the diet of Greek Byzantine populations reveal the importance of legumes and pulses as major staple food providing carbohydrates and proteins during the 6th and 15th century [46]. At the time of the Romans, or the medieval period, pulses were mixed with other grains yielding dark kinds of bread, leaving white bread to the rich and powerful. In many indigenous cultures and rural areas worldwide, legumes still substantially contribute to daily energy, protein and micronutrient intake as well as to soil fertility and rotating crop production [47,48]. Until the middle of the 19th century, in Germany, beans and lentils have been twice as important as potatoes and rice; however, since then, their consumption has decreased consistently (Figure 2). The technological improvement of mills, which enhance flour quality, and of meat production increasingly displaced pulses from the menu, and they received an image as a ‘poor man’s food’. In addition, beans and lentils, in contrast to white-flour products, are often associated with digestive constraints, such as flatulence and poor intestinal tolerance, which are objections and barriers that are still perceived at present. The long preparation time of dried pulses is an additional obstacle, now more than ever when convenient ready-to-heat/ready-to-eat alternatives are available. As a result, the consumption of legumes decreased massively from 1850 to 1970 from 20.7 to 0.9 kg/per capita and year [49]. This unfortunate development is still ongoing and has been observed in Western populations in general, which has raised scientific concerns [45,50,51]. This includes also the change of traditional dietary patterns, for example of the so-called Mediterranean Diet that is characterized amongst others by the relatively high proportion of legumes and pulses. Since the 1980′s the traditional Mediterranean Diet has attracted scientific interest due to its potential health promoting effects and reduction of cardiovascular mortality [52]. However, also in Mediterranean countries like Spain, the individual consumption of legumes and pulses has been decreasing for the last decades (from 20.2 g/day in 1991 to 11.9 g/day in 2010 and 13.9 g/day in 2013, respectively) below the Spanish dietary recommendation [53,54]. In line with other Western populations, traditional staples including pulses that deliver complex carbohydrates are being displaced increasingly by protein sources of animal origin as well as increasing fat and refined sugar intake [49,55].

Apart from being a traditional staple, dietary pulses may promote diet-related health as part of a balanced diet. Therefore, many attempts to raise public interest and promote legumes consumption have been made during the last years, e.g., along with attempts to generally increase fruit and vegetable consumption or the ‘International Year of Pulses’ campaign in 2016 by the Food and Agricultural Organization of the United Nations. The growing consumer demand for gluten-free, non-dairy or vegan foods led to the launch of various novel products that use either whole legumes or isolated fractions as food ingredients. However, these products often tend to be associated with a higher degree in food processing as the analysis from Australian food market launches suggests [56].

## 5. The Nutritional Benefit of Pulses Depends on the Applied Processing Techniques

Apart from carbohydrates, dried beans, peas and lentils contain high levels of protein (15–30%) and essential amino acids such as lysine and leucine, while cysteine and methionine are the limiting amino acids [57]. Due to the presence of antinutritive factors such as proteinase inhibitors, lectins, tannins and others, protein digestibility of raw unprocessed pulses is low [57,58]. Mild thermal and non-thermal processing significantly increases protein digestibility and diminishes the concentration and activity of these antinutritive factors [59,60,61,62,63]. Soaking and cooking of pulses result in the loss of many water-soluble compounds and reduces the protein content to approximately 10% [64]. Roasting or toasting, which is preferentially applied to obtain legume flours, yields edible products with higher protein concentration [57]. Germination and enzymatic processing may also increase protein concentration and digestibility of pulses [65,66]. Legumes and pulses are moreover a good source of mineral nutrients (iron, zinc), vitamins (folic acid, B vitamins), dietary fiber and secondary plant bioactives [57]. Phytic acid, present in pulses, chelates minerals (e.g., calcium, magnesium) and trace elements (e.g., iron, zinc, copper and manganese) and exerts adverse effects at higher concentration [67], but may be inactivated by phytase during germination or fermentation [63,64,68,69].

Therefore, several indispensable thermal and non-thermal processing techniques are applied yielding different types of pulse products from whole grains (e.g., cooked or canned beans) to food ingredients (e.g., flours or protein isolates). The supplementation of wheat and other cereal products with processed legumes and pulses increases protein concentration and quality and may contribute to a high nutritional quality in a plant-based diet [64,70].

Consumption of legumes and pulses is inversely associated with body weight [71], the incidence of type 2 diabetes (T2D) [72] and cancer mortality [73]. The postprandial glycaemic response to pulses is lower in healthy individuals and in type 2 diabetic patients, either ingested alone or in combination with other starchy staples [74,75,76,77]. Legumes provide cardiovascular health benefits (reviewed in [78]), although the results of cardiovascular disease (CVD)-related mortality are inconsistent [73,79]. Meanwhile, micronutrient status is superior in regular pulse consumers, especially that of thiamine, vitamin B6, folate, calcium, magnesium, iron, and zinc [50,51]. Therefore, legumes significantly contribute to nutritional quality in children at risk [80]. In fact, legume consumption has been identified as a protective dietary predictor of survival in long-lived elderly adults in different population groups [81].

## 6. National Dietary Recommendations with Respect to Legumes and Pulses Consumption

There is great variability in the beans, lentils and peas summarized as legumes, which are misconceived by many public health advocates, dieticians and consumers as solely a component of fruits and vegetables. For example, German consumers are suggested to eat ′five a day′ of fruits and vegetables, including seasonal fruits, leafy greens and others, as well as legumes. Nevertheless, nationwide nutrition monitoring demonstrates that the consumption of plant-based products is too low, while the level of consumed meat and meat products is too high, a relation that has been stable for decades [82]. Due to their nutritional profile and high protein concentration, legumes differ significantly from many other vegetables, and their dietary potential is often not fully exploited. In the USA, legumes are grouped with meat as ′protein-rich foods′, for example, in the National Heart, Lung, and Blood Institute’s Dietary Approaches to Stop Hypertension Eating Plan or Harvard’s Healthy Eating Pyramid but not in the US Department of Agriculture’s MyPyramid guideline [83]. In contrast, in Norway, Iceland or El Salvador, legume intake is not even specified in the recommendations, suggesting its minor role in national healthy eating guidelines. The recommendations for serving size and frequency, as much as the assignment to different food groups (either fruits and vegetables, protein-rich food or cereals and grains), vary among the national public dietary guidelines worldwide. Therefore, it is not surprising that dieticians and consumers easily lose track with current recommendations and are under-informed concerning the full dietary potential and possible health effects of legume consumption [84,85]. Table 1 gives examples of national dietary recommendations and guidelines worldwide, which can be viewed at the official website of the Food and Agriculture Organization of the United Nations [86]. Because official recommendation for daily legumes and pulses consumption are sometimes difficult to extract, we included the corresponding amount for cooked beans for better comparison calculated for those guidelines, where recommended intake was specified.

## 7. Future Directions

It may be difficult to predict the future and developments in food processing. However, given the current situation of the growing global population, especially in low- and middle-income countries, it seems plausible to propose that the efficient and sustained provision of high-quality nutritious food will be a major issue. It is conceivable that the next large transition in food processing will include the introduction of new biotechnologies to use alternative sources of protein and other nutrients and the development of novel innovative food. Such attempts have already been made considering microalgae as a source of protein, polyunsaturated fatty acids and many micronutrients [87], although it surely will take time to develop broad consumer acceptance of such innovations as daily foods. In contrast, the promotion of a side-lined (in modern times) but still traditional food, namely, legumes and pulses, seems more tangible and promising as a starting point. In addition to the beneficial effects on health, legumes are a low-cost dietary source of protein and micronutrients. Furthermore, health benefits, especially those related to obesity and type 2 diabetes prevention, may have substantial socioeconomic relevance [88]. Legumes are ecologically advantageous and exhibit a better nutrition carbon footprint score than does wheat [89]. The score reflects the nutritional quality per unit of environmental impact on the product level and calculates the nutrient balance score per unit of CO_2_ per serving. The partial replacement of refined wheat flour by local yellow pea flour in breakfast cereals, for example, impressively affects the nutrition carbon footprint score [89]. This last consideration further demonstrates that pulses and milled pulses may also be consumed in a similar manner as cereals and do not only serve as animal-based protein alternatives. As mentioned above, when consumed instead of common cereals such as wheat, barley or rice, micronutrient and fiber intake may be improved, and iron, folate and zinc insufficiency may be addressed, bypassing laborious industrial fortification. A minimum serving size of 100 g of prepared beans and lentils per day is assumed to improve the nutrient density of healthy and balanced diets, a level that is reached by regular consumers of pulses worldwide [90].

Due to the dietary and socioeconomic potential we suggest that legumes and pulses should comprise a separate food group in national dietary guidelines and that clear recommendations for daily serving sizes are provided (e.g., a minimum of 100 g of cooked pulses per day). From the consumer’s perspective, it may also be helpful to distinguish between different legumes (fresh or germinated seed), dry pulses and processed products (e.g., canned or used as ingredients) in the dietary recommendations. It makes a difference whether peas, beans and lentils are consumed as staple foods (along with potatoes, grains and others), as fresh vegetables, or as protein-rich foods like meat and dairy. In addition, the degree of food processing may affect nutritional quality and health benefits. We propose that legumes may be given more emphasis in the public, improving consumer education on the preparation of and variation in legumes and pulses. Since the consumption of pulses heavily depends on the knowledge of their preparation and culinary use, educational advice, recipes and cooking demonstrations may increase their familiarity, reduce potential objections and attract consumer interest again [85,91,92].

## Figures and Tables

**Table 1:** Official recommended dietary consumption of pulses and legumes in an international comparison (information retrieved from the Food and Agriculture Organization of the United Nations [86]). For the calculation of the daily amount retrieved from the respective guideline, it was assumed that 100 g of cooked pulses refer to 120 kcal, 1 cup equals 160 g of cooked beans, peas or lentils and one portion of dry yields two portions of cooked pulses.

Country	Official Name of the Guideline	Recommendations on Daily Consumption	Amount Calculated from the Guideline
Legumes and pulses comprise a separate food group and recommendation on daily intake is given			
Barbados	Food-Based Dietary Guidelines for Barbados	2–3 servings (1 serving = ½ cup of cooked beans or 2 ounces of legumes)	Up to 240 g of cooked pulses or 180 g of green legumes
Belize	Food-Based Dietary Guidelines for Belize (A Food Basket)	1–2 portion (73 kcal each portion)	40–80 g of cooked pulses
Brazil	Dietary Guidelines for the Brazilian Population 2014	One portion, 5% of total energy intake	Approximately 83 g of cooked pulses
Dominican Republic	The Mortar of Food and Nutrition	At least 1 cup	100–160 g of cooked pulses
Greece	National Nutrition Guide for Greek Adults	3–4 servings (including olives and nuts) from 22–23 in total (1 serving = 100 g of cooked beans)	Up to 400 g of cooked pulses
Jamaica	Food-Based Dietary Guidelines for Jamaica: Healthy Eating—Active Living	3 servings (73 kcal each serving) = ¾ cups	120 g of cooked pulses
Kenya	National Guidelines for Healthy Diets and Physical Activity	At least four times a week (1 serving is ½ cup)	At least 46 g of cooked pulses per day
Oman	The Omani Guide to Healthy Eating	1 serving (80 g)	80 g of cooked pulses
Portugal	Food Wheel Guide	1–2 portions (80 g of fresh legumes or 25 g of dry pulses per portion)	80–160 g of cooked pulses
Sierra Leone	Sierra Leone Food-Based Dietary Guidelines for Healthy Eating	Daily (1 serving is ½ cup)	80 g of cooked pulses
St. Kitts and Nevis	Food-Based Dietary Guidelines for St. Kitts and Nevis: The Sugar Mill	1–2 portions (73 kcal each portion = ¼ cup)	40–80 g of cooked pulses
Legumes and pulses comprise a separate food group, but recommendation on daily intake is not specified			
Antigua and Barbuda	Food-based Dietary Guidelines for Antigua and Barbuda (A Pineapple)	Not specified numerically	Approximately 1/8 of total amount of food
Qatar	Qatar Dietary Guidelines	Not specified numerically	Approximately 1/8 of total amount of food
South Africa	Food-Based Dietary Guidelines for South African	Not specified numerically	Approximately 1/8 of total amount of food
Spain	Eat Healthy and Move: 12 Healthy Decisions	At least 2–3 times per week; as source of carbohydrates every day	
Legumes and pulses are grouped with vegetables and fruits with specific recommendations on daily intake			
Austria	The Austrian Food Pyramid—7 Steps to Health	Up to 3 portions (1 portion = 150–200 g of cooked pulses)	Up to 600 g of cooked pulses
United States	Dietary Guidelines for Americans 2015—2020	1 ½-3 cups per week	At least 34 g of cooked pulses
Legumes and pulses are grouped with vegetables and fruits, but recommended daily intake is not specified			
Denmark	The Official Danish Dietary Guidelines	No specific information on pulses (at least 300 g of vegetables)	
France	The French National Nutrition and Health Program’s Dietary Guidelines.	At least 3 portions of vegetables, no specific information on pulses (at least twice the week)	
Germany	Ten Guidelines for Wholesome Eating and Drinking from the German Nutrition Society: The German Nutrition Circle	No specific information on pulses (at least 400 g of vegetables and fruits)	
Sweden	Find Your Way to Eat Greener, Not Too Much and to Be Active!	At least 500 g of vegetables and fruits, legume consumption is encouraged	
Legumes and pulses are grouped with starchy staples and specific recommended daily intake is given			
China	Food Guide Pagoda for Chinese Residents	50–150 g	50–150 g of cooked pulses
Costa Rica	Dietary Guidelines for Costa Rica: The Healthy Eating Circle	At least ½ cup of cooked pulses	At least 80 g of cooked pulses
India	Dietary Guidelines for Indians—A Manual	20–30 g of “raw” pulses each portion, 2-4 times a day	At least 75–150 g of cooked pulses
Legumes and pulses are grouped with starchy staples, but recommended daily intake is not specified			
Argentina	Dietary Guidelines for the Argentinian Population	Suggestion to combine pulses with cereals to replace meat in some plates	
Bolivia	Food-Based Dietary Guidelines for the Bolivian Population	3–10 portions of starchy staples including pulses	
Guatemala	Dietary Guidelines for Guatemala. Recommendations for Healthy Eating: The Family Pot	Suggestion to combine tortilla with pulses 1:2	
Honduras	Dietary Guidelines for Honduras. Tips for Healthy Eating: A Pot	Starchy staples with every meal	
Peru	Dietary Guidelines for the Peruvian Population	Approximately 1/3 of total food amount for the whole group	
Venezuela	Dietary Guidelines for Venezuela: The Food Spinning Top	Starchy staples are the major food group	
Legumes and pulses are grouped with other protein sources with specific recommendations			
Bangladesh	Dietary Guidelines for Bangladesh	1–2 servings as part of protein sources (1 serving = 30 g uncooked pulses) 6.5% of total energy	60–120 g of cooked pulses
Bulgaria	Food Based Dietary Guidelines for Adults in Bulgaria	At least twice a week (200–300 g/serving)	At least 57 g of cooked pulses
Cuba	Dietary Guidelines for the Cuban Population Over Two Years of Age	1 cup	160 g of cooked pulses
Georgia	Healthy Eating—The Main Key to Health	1–3 servings of the whole group (1 serving = 1/4 cup of cooked pulses), 150–200 g	Up to 120 g of cooked pulses
Ireland	Healthy Food for Life—The Healthy Eating Guidelines	2 servings of the whole group (1 serving = ¾ cup of pulses)	Up to 240 g of cooked pulses
Italy	Dietary Guidelines for Healthy Eating– Revision 2018	3 servings (1 serving = 150 g fresh legumes, 100 g tofu/tempeh or 50 g pulses)	Up to 450 g legumes and pulses
Japan	Dietary Guidelines for Japanese (Japan Food Spinning Top)	18–30 g of protein	200–300 g of cooked pulses
Lebanon	The Food-Based Dietary Guideline Manual for Promoting Healthy Eating in the Lebanese Adult Population (The Lebanese Cedar Food Guide)	5–6.5 servings of the whole group including nuts and seeds (1 serving = ¼ cup of cooked pulses)	200–260 g of cooked pulses
Mexico	Dietary and Physical Activity Guidelines in the Context of Overweight and Obesity in the Mexican Population	2 portions (1 portion = ½ cup of cooked pulses)	160 g of cooked pulses
Philippines	Nutritional Guidelines for Filipinos, 2012	4–5 servings of the whole group (1 serving = 1/3 cup of cooked pulses)	Up to 267 g of cooked pulses
Thailand	Food-Based Dietary Guidelines for Thai	6–12 tablespoons of the whole food group (1 tablespoon = ¼ tofu = 15 g)	Up to 180 g of cooked pulses
Turkey	Dietary Guidelines for Turkey: A Four-Leaf Clover	2 servings of the whole group (= 90 g pulses)	90 g of cooked pulses
Legumes and pulses are grouped with other protein sources such as meat, fish and eggs, but specific recommendations on daily intake are not given			
Benin	Benin’s Dietary Guidelines	No specific numerical recommendation	
Canada	Canada’s Food Guide	Whole food group comprises a “fourth of plate”	
Chile	Dietary Guidelines for the Chilean Population	At least twice the weak as sole protein source	
Colombia	Food-Based Dietary Guidelines for the Colombian Population Over 2 Years of Age: The Colombian Family’s Healthy Plate	At least twice the weak	
Ecuador	Food-Based Dietary Guidelines of Ecuador	No specific numerical recommendation, daily	
Iceland	Dietary Guidelines, for Adults and Children from Two Years of Age	Not specified	
Indonesia	Balanced Nutrition Guidelines	2–4 servings of the whole group	
Namibia	Food and Nutrition Guidelines for Namibia	No other specific recommendation than “regularly”	
Paraguay	Dietary Guidelines of Paraguay: The Paraguayan Nutritional Pot	Twice the week replacing meat, but also part of daily menu	
Republic of Korea	General Dietary Guidelines for Koreans: The Food Balance Wheels	3–4 servings of the whole food group	
United Kingdom	Eatwell Guide	No other specific recommendation than “some”	
Legumes and pulses are grouped with more than one food group with specific recommendations on daily consumption			
Australia	Australian Guide to Healthy Eating (Australian Dietary Guidelines)	2–3 servings as part of protein sources (1 serving = 150 g of cooked pulses) 5–6 servings of fruits and vegetables including pulses (1 serving = 75 g of cooked pulses)	At least 300 g of cooked pulses
Switzerland	The Swiss Food Pyramid	60–100 g of dry pulses as part of starchy staples 100–120 g of e.g., tofu as part of protein sources including meat, dairy, fish and eggs Fresh legumes (e.g., green peas) are grouped with vegetables	120–200 g of cooked pulses + 100–120 g tofu
Legumes and pulses are grouped with more than one food group, but recommended daily consumption is not specified			
Belgium	Practical Guidelines for Healthy Eating: The Food Pyramid for the French Population	Part of protein sources	
	The Food Triangle for the Flemish Population	Part of plant derived foods	
Bolivia	Food-Based Dietary Guidelines for the Bolivian Population	3–10 portions of starchy staples including pulses	
Netherlands	Dutch Dietary Guidelines	Part of protein sources (recommendation on pulses is “weekly”) Part of the group of vegetables (at least 250 g in total)	
Norway	Norwegian Guidelines on Diet, Nutrition and Physical Activity	Grouped with vegetables and protein sources	
